# Tartrate resistant acid phosphatase 5 (TRAP5) mediates immune cell recruitment in a murine model of pulmonary bacterial infection

**DOI:** 10.3389/fimmu.2022.1079775

**Published:** 2022-12-08

**Authors:** Lloyd Tanner, Jesper Bergwik, Ravi K. V. Bhongir, Manoj Puthia, Pernilla Lång, Mohamad N. Ali, Charlotte Welinder, Patrik Önnerfjord, Jonas S. Erjefält, Lena Palmberg, Göran Andersson, Arne Egesten

**Affiliations:** ^1^ Respiratory Medicine, Allergology & Palliative Medicine, Department of Clinical Sciences Lund, Lund University and Skåne University Hospital, Lund, Sweden; ^2^ Department of Dermatology and Venereology, Lund University and Skåne University Hospital, Lund, Sweden; ^3^ Department of Clinical Sciences Lund, Lund University and Skåne University Hospital, Lund, Sweden; ^4^ Division of Pathology, Department of Laboratory Medicine, Karolinska Institutet, Stockholm, Sweden; ^5^ Swedish National Infrastructure for Biological Mass Spectrometry (BioMS), Lund University, Lund, Sweden; ^6^ Molecular Skeletal Biology, Section for Rheumatology, Department of Clinical Sciences Lund, Lund University, Lund, Sweden; ^7^ Unit of Airway Inflammation, Experimental Medical Sciences, Lund University, Lund, Sweden; ^8^ Work Environment Toxicology, Institute of Environmental Medicine, Karolinska Institutet, Solna, Sweden

**Keywords:** NF-κB, immune cell recruitment, *Pseudomonas aeruginosa*, cystic fibrosis, TRAP5/ACP5, SPP1/OPN

## Abstract

**Introduction:**

During airway infection, upregulation of proinflammatory cytokines and subsequent immune cell recruitment is essential to mitigate bacterial infection. Conversely, during prolonged and non-resolving airway inflammation, neutrophils contribute to tissue damage and remodeling. This occurs during diseases including cystic fibrosis (CF) and COPD where bacterial pathogens, not least Pseudomonas aeruginosa, contribute to disease progression through long-lasting infections. Tartrate-resistant acid phosphatase (TRAP) 5 is a metalloenzyme expressed by alveolar macrophages and one of its target substrates is the phosphoglycoprotein osteopontin (OPN).

**Methods:**

We used a knockout mouse strain (Trap5-/-) and BALB/c-Tg (Rela-luc)31Xen mice paired with siRNA administration or functional protein add-back to elucidate the role of Trap5 during bacterial infection. In a series of experiments, Trap5-/- and wild-type control mice received intratracheal administration of P.aerugniosa (Xen41) or LPS, with mice monitored using intravital imaging (IVIS). In addition, multiplex cytokine immunoassays, flow cytometry, multispectral analyses, histological staining were performed.

**Results:**

In this study, we found that Trap5-/- mice had impaired clearance of P. aeruginosa airway infection and reduced recruitment of immune cells (i.e. neutrophils and inflammatory macrophages). Trap5 knockdown using siRNA resulted in a decreased activation of the proinflammatory transcription factor NF-κB in reporter mice and a subsequent decrease of proinflammatory gene expression. Add-back experiments of enzymatically active TRAP5 to Trap5-/- mice restored immune cell recruitment and bacterial killing. In human CF lung tissue, TRAP5 of alveolar macrophages was detected in proximity to OPN to a higher degree than in normal lung tissue, indicating possible interactions.

**Discussion:**

Taken together, the findings of this study suggest a key role for TRAP5 in modulating airway inflammation. This could have bearing in diseases such as CF and COPD where excessive neutrophilic inflammation could be targeted by pharmacological inhibitors of TRAP5.

## Introduction

During airway infection, pattern recognition receptors (PRRs) such as Toll-like receptors (TLRs) recognize conserved motifs on microbes, known as pathogen-associated molecular patterns (PAMPs). This results in activation of transcription factors that promote expression of proinflammatory cytokines and chemokines that attract immune cells such as neutrophils and inflammatory macrophages to the airways (1, 2). Alveolar macrophages play important roles in sensing invading bacteria through TLRs, resulting in activation of NF-κB, a key transcription factor promoting inflammation (3). Both macrophages and airway epithelium can upregulate their production of proinflammatory mediators upon activation of NF-κB, thereby orchestrating recruitment and activation of immune cells. In most cases, the inflammatory response helps eradicate bacteria and thereafter inflammation resolves. However, in chronic airway diseases such as cystic fibrosis (CF) and severe chronic obstructive pulmonary disease (COPD), there is a sustained neutrophil-rich inflammation complicated by both chronic and recurrent infections with the Gram-negative bacterial pathogen *Pseudomonas aeruginosa* (4, 5). Infection with *P. aeruginosa* is typically characterised by the detection of LPS and flagellin, PAMPs that engage TLR4 and TLR5 signalling cascades, respectively, initiating a pro-inflammatory response which facilitates further internalization and degradation of bacteria (6–11).

In this context, tartrate resistant acid phosphatase 5 (TRAP5), is a protein that is abundantly expressed in alveolar macrophages, immune cells that comprise the frontline response to lung infections (12, 13). TRAP5 is a cationic glycoprotein belonging to the class of binuclear metallophosphatases (14). It is synthesized as a single chain proenzyme, denoted 5a isoform (35 kDa) with low enzymatic activity, but upon posttranslational cleavage, two subunits (16-17 kDa and 20-23 kDa) are generated that are linked by disulphide bonds (the 5b isoform), having high enzymatic activity (15–18). TRAP5’s high abundance in alveolar macrophages suggests that it plays a vital role in pulmonary immunology, however, its precise function in this context is poorly understood, with high expression and activity in lung tissue of COPD and asthma patients (19). TRAP5 has additionally been shown to promote tumor metastasis in a number of solid cancers (20–22). In general, TRAP5a is considered a biomarker, reflecting disease activity in chronic inflammatory diseases, such as rheumatoid arthritis (23). Humans lacking TRAP5 suffer from the immuno-osseous dysplasia spondyloenchondrodysplasia (SPENCD), including short stature, autoimmune diseases, and a type I inflammation signature in blood. Interestingly, two out of eight investigated individuals had a history of recurring infections, including lobar pneumonia (24).

A protein which plays a tight-knit role with TRAP5 is osteopontin (OPN), a negatively charged phosphoglycoprotein, that is secreted into numerous body fluids and expressed by a variety of cells such as, osteoclasts, immune cells, and epithelial cells (25). OPN is the main substrate for the phosphatase activity of TRAP5, being critical for macrophage and osteoclast migration on immobilized OPN (14, 19, 26). It is involved in immune regulation and inflammation, where it acts as an important proinflammatory mediator and as a chemoattractant for macrophages and neutrophils (27–29). OPN is elevated in airways during inflammatory diseases such as cystic fibrosis (CF) and chronic obstructive pulmonary disease [COPD (30, 31)]. However, the precise role of TRAP5 during CF-related infections is unknown. In this study, the aim was to investigate the role of TRAP5 during airway inflammation resulting from bacterial infection.

## Materials and methods

### Study design

The goal of this study was to assess the role of TRAP5 in immune cell recruitment during lung infection. Subsequent *in vivo* murine studies using intratracheally-administered *Pseudomonas aeruginosa* and LPS were chosen as well-established and relevant models of experimental lung infection. Sample sizes were calculated by power analysis based on previous experience, feasibility, and to conform to the ARRIVE guidelines. For initial lung infection experiments testing the viability of TRAP5 as a drug target in siRNA experiments, *n* =4 mice per group were used to achieve statistical significance. For further confirmatory experiments, *n* ≥ 5 to 7 mice per group were used. Mice were randomly assigned to treatment groups. Downstream analyses were conducted with the investigator blinded to the treatment groups, and no animals were excluded as outliers from the reported dataset. All *in vitro* and *in vivo* experiments were performed in two to four technical replicates. Human resected lung sections were obtained with informed consent, with a statistically significant *n=*4 samples used for disease and healthy control samples.

### Statement of ethics

All animal experiments were approved by the Malmö‐Lund Animal Care Ethics Committee (M3802-19). Lung tissue was obtained after written informed consent, approval by the Regional Ethical Review Board in Lund (approval no. LU412-03) and at Karolinska Institutet, Solna (95–347), and performed in accordance with the Declaration of Helsinki as well as relevant guidelines and regulations.

### *In vitro* human macrophage recruitment assay

Human leukocyte concentrate was purified using positive CD14+ magnetic bead (Miltenyi Biotech, Lund, Sweden) selection. Macrophages were stimulated for 4 days using hMCSF, followed by TRAP5 knockdown using TRAP5-targeting or non-targeting control siRNA (25nM). Cells were then utilized in a transwell assay, with membranes coated with either bovine collagen (ThermoFisher, MA, USA) or hOPN (R&D Systems, MN, USA). Cells were then stimulated with RANKL (10 nM) and allowed to migrate for 72h. Cells in the basal well were measured using flow cytometry by analyzing CD14+ cells.

### Immunoblotting

Total protein concentrations of lung homogenate lysates were determined and SDS-PAGE was performed using Mini-PROTEAN^®^ Precast Mini PAGE Gels (Bio-Rad, Hercules, CA). Trans-Blot Turbo Mini 0.2 µM PVDF Transfer Packs (Bio-Rad) were used for transferring of proteins to the PVDF membranes. Membranes were blocked for 3h at RT and incubated with primary antibodies (rabbit anti-human TRAP5b (provided by Dr. Göran Andersson), rabbit polyclonal to TRAP5 (Cat #PA5-116970; Invitrogen, MA, USA), and rabbit anti-mouse GAPDH (Cat #MA5-15738-D680; Invitrogen 1:500) in blocking buffer. After washing with PBS-Tween, membranes were incubated with secondary antibodies (Alexa Fluor 488-conjugated goat anti rabbit/mouse (Invitrogen, Carlsbad, CA) for 1h RT. Imaging of blots was preformed using a ChemiDoc system (Bio-Rad) followed by quantification with densitometry normalized to GAPDH.

### Characterization of OPN phosphorylation

Characterization of OPN phosphorylation was carried out essentially as described (32). Briefly, recombinant human OPN derived from HEK293 cells (Peprotech, Rocky Hill, NJ) and expressed in *E. coli* (Abcam) with protein purities of ≥ 97% and ≥ 90% respectively according to the manufacturers were digested either with trypsin (Sequencing Grade Modified Trypsin, Part No. V511A; Promega, Madison, WI) at a ratio of 1:50 to the samples and incubated overnight at 37°C or with thermolysine (Part No. V400A; Promega) at a ratio of 1:20 and incubated at 85°C for 5 hrs. The digestion was stopped by addition of 5 µL 10% trifluoroacetic acid. The peptides were concentrated using refrigerated vacuum centrifugation (Speed Vac) to dryness and resolved in 2% ACN, 0.1% TFA.

### Mass spectrometry

Peptides were analyzed using a quadrupole Orbitrap benchtop mass spectrometer (QExactive) (Thermo Fisher Scientific, Waltham, MA) equipped with an Easy nano-LC 1000 system (Thermo Scientific). Separation was performed on 75 μm × 25 cm capillary columns (Acclaim PepmapTM RSLC, C18, 2μm, 100Å; Thermo Fischer Scientific). A spray voltage of +2000 V was used with a heated ion transfer setting of 275°C for desolvation. The on-line reversed-phase separation was performed on an Easy nano-LC 1000 system using a flow rate of 300 nl/min and a linear binary gradient from 3% solvent B for 4 min to 9% solvent B, then to 25% solvent B for 32 min, then to 35% for 5 min and finally isocratic 90% solvent B for 10 min. An MS scan (400–1800 m/z) was recorded in the Orbitrap mass analyzer set at a resolution of 70,000 at 200 m/z, 3×10^6^ automatic gain control (AGC) target and 100 ms maximum ion injection time. The MS was followed by data-dependent high energy collision-induced dissociation (HCD) MS/MS scans at a resolution of 15,000 on the 15 most intense multiply charged ions at 2 × 10^4^ intensity threshold, and dynamic exclusion enabled for 20 seconds.

### Data analysis

MS/MS spectra were searched with PEAKS (version 8.5) against UniProt Homo Sapiens (UP000005640, Organism ID 9606). The precursor tolerance and the fragment tolerance were set to 15 ppm and 0.05 Da, respectively. Trypsin or no enzyme (for thermolysine) were selected with one missed cleavage allowance, phosphorylation of serine, threonine and tyrosine, methionine oxidation and deamidation of aspargine and glutamine were treated as dynamic modification.

### Bronchoalveolar fluid from individuals exposed to endotoxin-containing organic dust

BALF from eight healthy individuals (6 men and 2 women) were obtained before and 24 hours after a three hour of exposure in a swine confinement facility. Details of person enrollment, study protocol and BALF sampling techniques are described elsewhere (26). TRAP5a and TRAP5b were measured by ELISA (33).

### *Pseudomonas aeruginosa* killing assay

*P. aeruginosa* (strain PAO1) was grown to mid-log phase in TH broth (Becton, Dickson and Company, Franklin Lakes, NJ) at 37°C, washed, and diluted in incubation buffer (10 mM Trizma base, (Sigma-Aldrich, St Louis, MO) + 5 mM Glucose, (VWR, Stockholm, Sweden)) to make a 1% bacterial stem solution. First, dose-response curves for the bactericidal activity of the antimicrobial peptides (AMPs): hBD-3 (Abcam), SLPI (Abcam), and LL37 (Schaefer, Copenhagen, Denmark) were obtained. Thereafter, experiments were performed where various concentrations of OPN expressed in HEK293 cells (pOPN; Peprotech) and *E. coli respectively* (dOPN; Abcam) were preincubated (20 min) with the AMPs before being added to 35 µL of a 0.001% bacterial solution to a final volume of 50 μL. Samples were incubated at 37°C for 1 h, before 450 μL of ice cold Tris-Glucose buffer was added to each sample. Serial dilutions were plated on TH agar plates, followed by incubation at 37°C overnight, after which colony forming units were quantified.

### Surface plasmon resonance

The affinity between dephosphorylated or phosphorylated OPN and the AMPs hBD3, SLPI, and LL37 was assessed using SPR analysis. In different flow cells, hBD3, SLPI, and LL37 were immobilized as surface ligands at 50 μg/mL in 10 mM sodium acetate (pH 5.5) for 5-10 min. 1M ethanolamine-HCL (pH8.5) was injected for 6-7 min to deactivate excessive reactive groups on the sensor chip surface. dOPN (non-phosphorylated) and pOPN (phosphorylated) were added to separate sensor chip surfaces at 10 μL/min in HBS-EP buffer at indicated concentrations. All buffers, reagents, and equipment were purchased from GE Healthcare. Analyses were performed using the BIAcore X100 instrument (GE Healthcare, Chicago, IL) using sensor chip technology at 25°C in a degassed HBS-EP buffer (10 mM HEPES, 150 mM NaCL, 3 mM EDTA, 0.05% (v/v) surfactant p20, pH 7.4) with a constant flow rate of 10 μL/min. Sensorgrams were analyzed using BIA Evaluation 4.1 software (GE Healthcare). Following x/y normalization, blank curves from vehicle flow cell were subtracted from the data and the dissociation (*k_d_*) and association (*k_a_*) constants were calculated using a Langmuir model.

### Collection and preparation of human lung tissue samples

Control lung tissues were obtained from otherwise healthy, non-smoking patients undergoing surgery for lung cancer (four donors) while CF lung tissues were obtained from patients suffering from CF, undergoing lung transplantation (four donors) (**Supplemental Table I**). The tissues were formalin-fixed, embedded in paraffin, and sectioned at 4 µm thickness. After dewaxing and rehydration, epitope retrieval was performed using a PT-link Rinse Station (PT109; Agilent, Santa Clara, CA) with a low pH retrieval buffer (K8005; Agilent) according to a protocol provided by the manufacturer. Thereafter, the samples were washed twice for 3 min in TBS supplemented with 0.05% Tween-20 (used for all subsequent washes unless stated otherwise). The samples were delineated with an ImmEdge pen (H-4000; Vector Laboratories, Burlingame, CA) followed by blocking peroxidase-activity (K8010; Agilent) for 10 min at RT and then washed. After this, the samples were stained *via* immunohistochemistry and Navinci (described below).

### Detecting physical association between TRAP5 and OPN using a proximity-ligation assay

A partly modified proximity ligation protocol (Navinci, https://www.navinci.se; Moleculink AB; Uppsala, Sweden) was applied to detect close physical association between OPN and TRAP5 in lung tissues collected and prepared as described above. After blocking peroxidase-activity, tissue sections were incubated using a blocking mixture (1 µg/µl BSA; 1.65 µg/ml Salmon sperm DNA; 2 mM L-Cysteine (cat. # C7352; Merck); 5 µg/ml Avidin (cat. # 21121; Thermo Fisher Scientific); 0.5 µg/µl Goat serum (cat. # X0907; Agilent), at 4°C for 4 h. Subsequently, the sections were incubated with diluted primary antibodies (rabbit anti-OPN serum at 1:5 000 and a murine monoclonal anti-TRAP5 antibody (Hycult Biotech, Uden, The Netherlands) at 1:300 (0.33 µg/ml) in an antibody-diluent supplied by the manufacturer, supplemented with the same blocking reagents as the blocking mixture, apart from excluding avidin, and allowing the incubation to proceed at 4°C overnight. Following this, the samples were incubated with secondary probes according to the protocol supplied by the manufacturer, supplemented with 0.1 mg/ml Biotin (cat. # 29129; Thermo Fisher Scientific), and thereafter the digestion and ligation steps were carried out according to the manufacturer’s protocol. Avidin and biotin reagents blocked non-specific binding, allowing the later use of a streptavidin-HRP conjugate. Next, the amplification step was executed using the buffer “Buffer C Biotin” with an added custom compaction oligonucleotide (Integrated DNA Technologies, Coralville, IA) with the sequence:

5’-GAAAGAGTGTCTAGTTCTGTCAAAGAAAGAGTGTCTAGTTCTGTCmUmUmUmU-3’ at 25 nM for signals of more distinct appearance and the incubation time was extended to 260 min. To allow a chromogenic read-out, the samples were incubated with a streptavidin-HRP conjugate (cat. # S2438; Merck, Kenilworth, NJ) at 3.2 µg/ml in a TBS buffer containing 0.25 mg/ml BSA, at RT for 60 min. After washing with TBS for 5 min, the sections were incubated with VinaGreen (cat. # BRR807AH; Biocare Medical, Pacheco, CA) at RT for 10 min, rinsed in distilled H_2_O, followed by dipping in Mayer’s Hematoxylin (cat. # 01820; Histolab, Askim, Sweden) at RT for 30 sec and then left in flowing cold tap water for 5 min. Finally, the sections were rinsed in distilled H_2_O, dried at 60°C for 20 min and finally mounted with Pertex (cat. # 00840-05; Histolab) on cover slips before imaging.

### Animals

10-12-week-old male wild-type C57Bl/6 mice (Janvier, Le Genest-Saint-Isle, France), Trap5^-/-^, and male BALB/c-Tg(NF-κB-RE-Luc)-Xen reporter mice (Taconic Biosciences, Albany, NY) were housed at least 2 weeks in the animal facility at the Biomedical Service Division at Lund University before initiating experiments and were provided with food and water *ad libitum* throughout the study. Trap5^-/-^ mice (generously provided by Dr. Thorsten Schinke, University Medical Center Hamburg-Eppendorf, Hamburg, Germany) have been described elsewhere and their genotype was confirmed before this study by whole genome sequencing [**Supplementary Figure 1** (34)]. Mice were randomly allocated into experimental groups for each experiment. In the *Pseudomonas aeruginosa* administration experiments mice were divided as follows (n=5-8 per group): (i) wild-type mice intratracheally (i.t.)-administered *Pseudomonas aeruginosa* Xen-41 (5x10^7^ CFU/mL), (ii) wild-type mice intratracheally (i.t.)-administered saline, (iii) Trap5^-/-^ mice intratracheally (i.t.)-administered *Pseudomonas aeruginosa* Xen-41 (5x10^7^ CFU/mL), (iv) Trap5^-/-^ mice intratracheally (i.t.)-administered saline. For the siRNA administration experiments in male BALB/c-Tg(NF-κB-RE-Luc)-Xen reporter mice (n=4 per group) (i) mice were intratracheally administered Trap5-targeting or non-targeting siRNA, which was then followed by intratracheal administration of LPS (4h later). In the add back experiments, mice were grouped as follows (n=4-6): (i) wild-type mice intratracheally (i.t.)-administered *Pseudomonas aeruginosa* Xen-41 (5x10^7^ CFU/mL), Trap5^-/-^ mice were intratracheally (i.t.)-administered (ii) vehicle, (iii) recombinant human TRAP5 expressed in *E. coli*, (iv) and (v) high (8ng/mL) and low (2 ng/mL) doses of recombinant TRAP5 expressed in HEK293 cells (Abcam, Cambridge, UK), with mice subsequently intratracheally administered *Pseudomonas aeruginosa* Xen-41 (5x10^7^ CFU/mL). Lipopolysaccharide (LPS) was obtained from Sigma-Aldrich.

### *In vivo* imaging

An IVIS *in vivo* imaging system was used for the longitudinal evaluation of NF-κB activation (PerkinElmer, Waltham, MA). Mice were injected intraperitoneally with 100 μl of d-luciferin (150 mg/kg body wt; PerkinElmer) 15 min before the imaging. Bioluminescent signals from bacterial infection were acquired and quantified using Living Image 4.0 software (PerkinElmer).

### Bronchoalveolar lavage fluid collection

BAL was performed with a total volume of 1 ml PBS containing 100 μM EDTA. BALF was collected in Eppendorf tubes on ice, with aliquots made for flow cytometry, cytospin differential counts, and an aliquot transferred to -80°C for multiplex cytokine analysis. Cytospin preparations of cells were stained with modified Wright-Giemsa stain (Sigma-Aldrich). Murine TRAP5 was measured in BALF using ELISA (Abcam).

### Collection of lung tissue

Right lungs were collected in Eppendorf tubes on dry ice and stored at -80°C. The snap-frozen lungs were thawed and homogenized in tissue protein extraction reagent (T-PER) solution (Thermo Fisher Scientific) containing protease inhibitor (Pefabloc SC; Sigma-Aldrich) at a final concentration of 1 mM. Lung homogenates were centrifuged at 9,000 x *g* for 10 min at 4°C, and the supernatants were collected for multiplex analysis. Left lungs were collected in Histofix (Histolab) and submerged in 4% buffered paraformaldehyde solution.

### Bacterial plating

Mice were sacrificed post-infection, and organs (lung, liver, spleen, heart, and kidney) were harvested and plated on TH agar plates to determine the degree of bacterial dissemination (reported as CFU/mg of tissue).

### Mouse ACP5 ELISA

Mouse lung homogenate (preparation described above) was analyzed using a mouse ACP5/TRAP5 ELISA (LS-F28187; LSBio, Washington, USA). Samples were prepared according to the manufacturer’s instructions and read (450nm) using a VICTOR 1420 Multilabel plate reader (PerkinElmer).

### H&E staining of lung tissue

Mouse left lungs were fixed in Histofix (Histolab), paraffin-embedded and sectioned at 3 µm. The tissue sections were placed on slides (Superfrost Plus; Thermo Fisher Scientific) and deparaffinized in serial baths of xylene and ethanol followed by staining using Mayer hematoxylin and 0.2% eosin (Histolab). The stained slides were imaged using an Aperio CS2 image capture device.

### Flow cytometry

Flow cytometry was carried out using a BD Accuri C6 Plus (BD Biosciences, Franklin Lakes, NJ). The washed cells were incubated with Fixable Viability Stain 510 (FVS510; BD #564406) to differentiate live and dead cells. Cells were washed with Stain buffer 1x (BD #554656) and incubated with Lyse Fix 1x (BD #558049 (5x)). Fixed cells were washed with stain buffer and aliquoted into two samples incubated with either anti-CD11b (BD553312), anti-CD11c (BD558079), anti-Ly6G (BD551461), or anti-CD11b, anti-Ly6C (BD 553128), anti-CD115 (BD 565249) antibodies.

### Multiplex cytokine analysis

For the detection of multiple cytokines in BALF, plasma, and lung homogenate, the Bio-Plex Pro mouse cytokine assay (23-Plex Group I; Bio-Rad) was used on a Luminex-xMAP/Bio-Plex 200 System with Bio-Plex Manager 6.2 software (Bio-Rad). A cytometric magnetic bead-based assay was used to measure cytokine levels, according to the manufacturer’s instructions. The detection limits were as follows: Eotaxin (4524.58-1.23 pg/mL), GCSF (99318.6-7.3 pg/mL), GMCSF (6310.48-3.91 pg/mL), IFN-γ (16114.01-0.87 pg/mL), IL-1α (10055.54-0.54 pg/mL), IL-1β (31512.04-1.75 pg/mL), IL-2 (19175.48-1.24 pg/mL), IL-3 (7514.5-0.44 pg/mL), IL-4 (5923.58-0.34 pg/mL), IL-5 (12619.59-0.78 pg/mL), IL-6 (9409.63-0.68 pg/mL), IL-9 (64684.09-2.41 pg/mL), IL-10 (77390.75-4.18 pg/mL), IL-12p40 (144560.15-18.62 pg/mL), IL-12p70 (78647.56-4.81 pg/mL), IL-13 (197828.67-11.16 pg/mL), IL-17 (8727.85-0.51 pg/mL), KC (23001.9-1.4 pg/mL), MCP-1 (393545.52-10.01 pg/mL), MIP-1α (14566.62-0.63 pg/mL), MIP-1β (7023.87-0.34 pg/mL), RANTES (19490.48-4.61 pg/mL), and TNF-α (74368.54-51.69 pg/mL). Cytokine measurements for lung homogenate samples were corrected for total protein concentration using a Pierce™ BCA Protein Assay Kit (Thermo Fisher Scientific).

### OPAL staining

Sections (3 µm thick) were adhered to SuperFrost^®^ Plus (Menzel Gläser, Braunschweig, Germany) and processed for OPAL multi-immunohistochemistry. Sections were stained using the Automated Opal 7-Color IHC Kit (Akoya Bioscience, Menlo Park, CA) in a Bond RXm (Leica, Wetzlar, Germany). Primary antibodies were MHC class II (Abcam) 1:200, anti CD206 1:350 and anti-PA 1:100. After staining, sections were mounted using VECTASHIELD^®^ Antifade Mounting Medium (Vector laboratories, Burlingame, CA).

### Multispectral scanning documentation and analysis

Multispectral images (MSI) were obtained from one section from 9 animals in each group, in total 9 sections using Vectra 3 Automated Quantitative Pathology Imaging System (Akoya Biosciences). The total number of MSI/group were WT uninfected n = 296, WT infected n = 372, KO uninfected n = 373, KO infected n = 337. These images were pooled in each group to represent a fictive lung in each group. InForm Tissue Analysis Software (Akoya Biosciences) was used to segment the tissue and to perform cellular phenotyping. Analysis of phenotyping data was then further processed using RStudio Team (2020) RStudio: Integrated Development for R. (RStudio, PBC, Boston, MA) URL http://www.rstudio.com/ together with the add-in PhenoptrReports and phenoptr (Akoya Biosciences).

### Statistical analysis

In this study, groups of three or more mice were compared using one-way analysis of variance (ANOVA) with Dunnett’s *post hoc* test. In experiments using two groups, results were compared using unpaired *t* test with Welch’s correction. Results in this study are displayed throughout as mean ± SEM. Statistical testing was carried out using GraphPad Prism 9.1.1 (GraphPad Software, San Diego, CA) with statistical significance defined as *P* < 0.05.

## Results

### TRAP5 deficiency results in decreased macrophage recruitment

**Figure 1:**
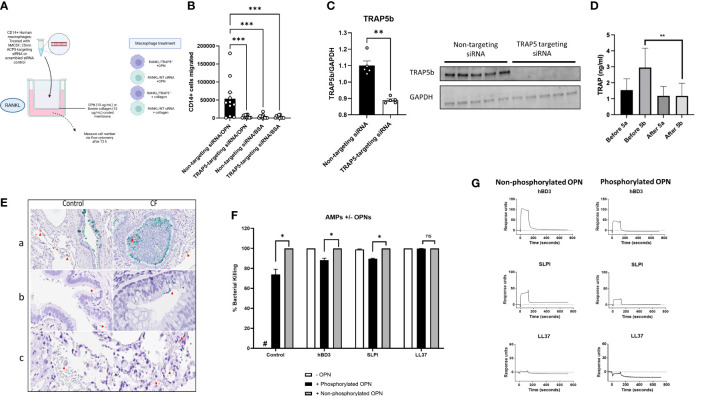
Immune cell recruitment and TRAP5 expression in patient samples. **(A)** Human leukocytes were treated with rhM-CSF for 5 days. Cells were then transfected with TRAP5-targeting siRNA or scrambled siRNA control. **(B)** Cells were added to a transwell assay and CD14+ cells were measured in the basal well after 72h of RANKL stimulation. Statistical analysis was conducted using one-way ANOVA with a Dunnet’s *post hoc* test (***P<0.001). **(C)** Immunoblotting for TRAP5b from human macrophage cell lysates. **(D)** TRAP5a and 5b levels in human BALF collected from patients exposed to swine dust. TRAP isoform levels were determined *via* enzyme-linked immunosorbent assay (ELISA). Results represent the mean and standard deviation of all the samples run. Statistical analysis was performed using Student’s t-test (** P<0.01). **(E)** Immunohistochemical detection of TRAP5a/OPN in human lung tissue using a proximity-ligation assay (Navinci), with interactions between the two molecules shown in green. Images show (a) OPN and TRAP5a in small airways, (b) in alveolar macrophages and (c) capillaries. The red arrows show the TRAP5a positive cells. Image (a): 40x magnification. Images (b, c): 60x magnification. **(F)** *Pseudomonas aeruginosa* killing assay showing bacterial killing following hBD3, SLPI, and LL37 (0.1 μM) following incubation with either dOPN or pOPN. Statistical analysis was conducted using one-way ANOVA with a Dunnet’s *post hoc* test (*P <0.05). **(G)** SPR sensorgrams illustrating interactions between hBD3, SLPI, LL37, and dOPN/pOPN. NS-not significant.

Osteoclast migration has previously been shown to be influenced by TRAP5 expression, predominantly through TRAP5 dephosphorylation of osteopontin (OPN (14, 35);). Macrophage recruitment was investigated using human-derived macrophage cells treated with TRAP5-targeting siRNA (**Figures 1A, B**). Macrophage migration on OPN in cells receiving TRAP5-targeting siRNA was significantly decreased following 72h of transwell incubation compared to non-targeting siRNA control. Immunoblotting of human macrophage cells treated with RANKL for 72h and TRAP5 siRNA displayed significantly reduced levels of the TRAP5b isoform (**Figure 1C**). To investigate the effects of an inflammatory stimulus on TRAP5 levels, healthy individuals were exposed to inflammatory-inducing swine dust. Noticeably reduced BALF levels of TRAP5b were seen, with levels of TRAP5a unchanged following exposure (**Figure 1D**). To investigate TRAP5a and OPN co-localization within similar lung areas, a Proximity Ligation Assay (PLA) was used. Our results demonstrate the colocalization of OPN and TRAP5a in the small airways, to a greater degree in the CF lung compared to healthy control tissue (**Figure 1E**). Specific staining was seen in both goblet cells and macrophages. This data suggests these two proteins interact in the small airways and blood, leading to the de-phosphorylation of OPN by TRAP5, particularly prevalent within the CF disease-state.

### TRAP5 dephosphorylation of OPN increases bacterial killing by AMPs

The phosphoglyoprotein OPN is a target of TRAP5 phosphatase activity. To determine whether the phosphorylation status of OPN affects bacterial killing of *P. aeruginosa* by common antimicrobial peptides (AMPs) of the airways, we assessed human β-defensin-3 (hBD3), secretory leukocyte protease inhibitor (SLPI), and LL37, incubated with OPN (dephosphorylated (dOPN) and phosphorylated (pOPN)), respectively. The phosphorylation pattern of OPN expressed in HEK293 cells and *E. coli* were investigated using mass spectrometry as described (32) (**Supplementary Figure 2**). Significant decreases in *P. aeruginosa* killing following addition of pOPN but less in the case of dOPN were seen (**Figure 1F**). We further tested whether phosphorylation of OPN affected binding antimicrobial peptides (**Figure 1G**). AMPs were tested for binding to either dOPN or pOPN, with significant decreases seen for hBD3 (KD (M): 6.496E^-9^ for pOPN versus 8.383E^-8^ for dOPN) and SLPI (KD (M): 6.251E^-9^ for pOPN versus 4.652E^-6^ for dOPN). Therefore, dephosphorylation of OPN favors decreased binding of AMPs.

### TRAP5 deficiency significantly decreases murine bacterial sequestration and clearance

**Figure 2:**
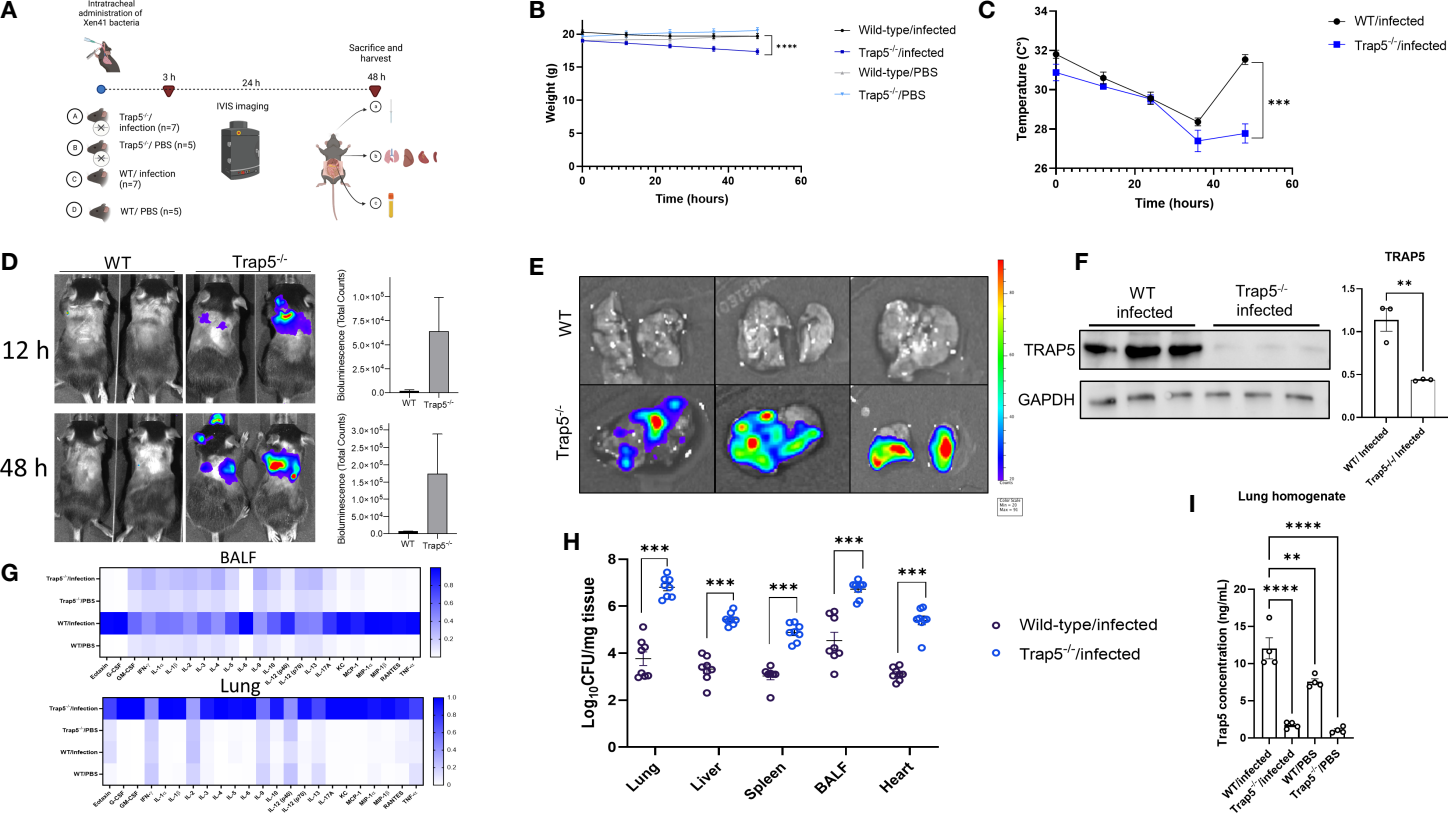
Immune cell recruitment following bacterial infection is impaired in Trap5 knockout mice. **(A)** C57BL6/J mice (both Trap5^-/-^and wild-type (WT)) were infected with Xen41 bioluminescent bacteria for 48h. **(B, C)** Murine weight and surface body temperature was measured over the course of the experiment with Trap5^-/-^ mice losing significantly more weight and total body temperature after 48h compared to WT mice. Statistical analysis was performed using Student’s t-test. ***P<0.001 and ****P<0.0001. **(D)** Murine infection was monitored using IVIS *in vivo* imaging with bioluminescence quantified alongside. **(E)** Murine lungs were removed and viewed using IVIS. **(F)** Murine lung homogenate was assessed for TRAP5 expression with quantification alongside. Statistical analysis was performed using student’s t-test. **P  <  0.001. **(G)** Bioplex cytokine array was used to assess murine lung homogenates and BALF with heatmap showing mean normalized values (white-low value; blue-high value). **(H)** Organ homogenates were assessed for bacterial growth. Statistical analysis was conducted using one-way ANOVA with a Dunnet’s *post hoc* test (***P <0.001). **(I)** TRAP5 concentrations were measured in lung homogenate using ELISA. Statistical analysis was conducted using one-way ANOVA with a Dunnet’s *post hoc* test (**P <0.01; ****P <0.0001).

To further elucidate whether TRAP5 plays a role during infection-induced inflammation, Trap5^-/-^ mice (whole genome sequenced, **Supplementary Figure 1**) and wild-type (WT) controls were intratracheally administered bioluminescent *Pseudomonas aeruginosa* (PAO1 Xen41) and monitored for 48h (**Figure 2A**). Murine body weight and body temperature were measured over the course of the experiment with Trap5^-/-^ infected mice displaying more weight loss and temperature decreases compared to the WT control mice (**Figures 2B, C**). IVIS monitoring of bacterial specific-bioluminescent signal in live mice resulted in increased lung-specific signal in the Trap5^-/-^ mice compared to the WT controls (**Figure 2D**).

Murine lungs were removed and viewed using the IVIS which showed more signal in the Trap5^-/-^ lungs compared to the WT controls (**Figure 2E**). Immunoblotting of murine lung homogenates confirmed the significant decreases in TRAP5 expression in the Trap5^-/-^ mice compared to the WT controls (**Figure 2F**). Analysis of cytokines in the BALF and lungs revealed significantly increased cytokines in the lungs of the Trap5^-/-^ mice compared to the WT controls. Furthermore, significantly increased levels of cytokines were seen in the BALF of the WT controls compared to the Trap5^-/-^ mice (**Figure 2G** and **Supplementary Figures 3**–**5**). More specifically, pro-inflammatory cytokines G-CSF, IL-6, KC, and MIP-1β were decreased in the BALF and increased in the lung tissue of Trap5^-/-^ mice compared to WT controls. Organ homogenization and bacterial plating further supported the results of these readouts, with Lung and BALF bacterial counts in the Trap5^-/-^ mice representing the highest bacterial concentrations within the mouse (**Figure 2H**). Bacterial dissemination was significantly higher in spleen, liver, and heart tissues of Trap5^-/-^ mice compared to corresponding organs in WT mice. Finally, TRAP5 concentrations were measured in the lung tissues of mice, with significantly more TRAP5 in the lung tissue of WT mice as expected, and with significant increases in TRAP5 levels in WT mice following infection (**Figure 2I**).

### Immune cell recruitment is significantly decreased following TRAP5 knockout

**Figure 3:**
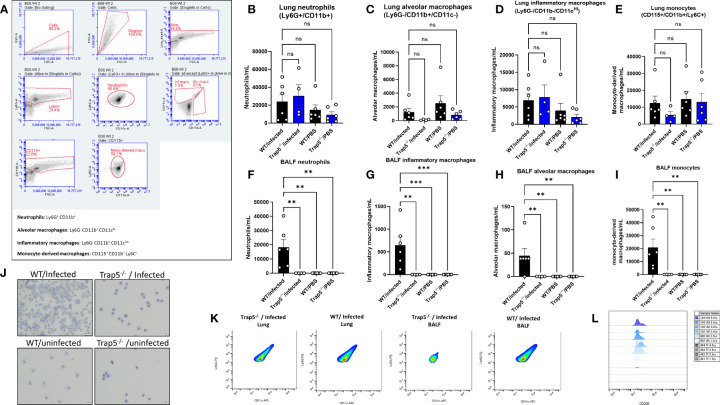
Immune cell recruitment following bacterial infection. **(A)** Representative gating of murine cells is shown from Trap5^-/-^ and WT infection experiments. **(B–E)** Murine lung cells and **(F–I)** BALF cells were quantified per mL of tissue or fluid. Murine neutrophils (live dead^+^/Ly6G^+^/CD11b^+^), alveolar macrophages (live dead^+^/Ly6G^-^/CD11b^+^/CD11c^-^), inflammatory macrophages (live dead^+^/Ly6G^-^/CD11b^+^/CD11c intermed), and monocytes (CD115^+^/CD11b^+^/Ly6C^+^) were measured using flow cytometry. Statistical analysis was conducted using one-way ANOVA with a Dunnet’s *post hoc* test (**P <0.01; ***P <0.001). **(J)** Representative cytospin images of murine BALF are presented. **(K)** Representative plots showing the differences in neutrophil populations in the lung and BALF samples, with CD206+ signal presented in the monocyte population in **(L)**. NS-not significant.

To further explain the decreases in bacterial killing in the absence of TRAP5 expression, murine BALF and lung immune cells were examined, usingflow cytometry gating scheme shown in **Figure 3A**. Trap5^-/-^ murine lung tissues showed similar numbers of immune cells to WT lung tissue, with minor decreases seen in alveolar macrophage numbers in the Trap5^-/-^ mice (**Figures 3B–E**). However, Trap5^-/-^ mice displayed significantly reduced neutrophil, alveolar macrophage, inflammatory macrophage, and monocyte recruitment to the BALF following infection (**Figures 3F–I**). Confirmatory microscopy revealed increased BALF immune cell recruitment following infection in the WT group compared to significantly fewer cells in the Trap5^-/-^ infection group (**Figure 3J**). Furthermore, populations of neutrophils (**Figure 3K**) and CD206+ monocytes (**Figure 3L**) were significantly decreased in the Trap5^-/-^ group versus the WT control group in the BALF. This strongly supports that the lack of immune cell recruitment to the BALF is the basis for increased vulnerability to bacterial infection seen in previous experiments.

### Distribution of macrophages in infected lung tissue and immune cell recruitment

**Figure 4:**
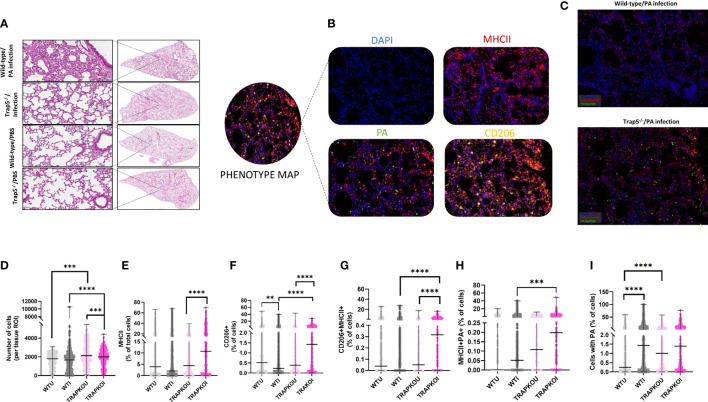
Trap5 depletion increases lung specific immune cell content. **(A)** Haemotoxylin & eosin staining of wild-type and Trap5^-/-^ murine lungs. **(B)** Phenotype mapping of multispectral imaging (MSI) showing representative individual stains for each antibody used. **(C)** Multispectral images (MSI) were obtained from 9 animals in each group using Vectra 3 Automated Quantitative Pathology Imaging System (Akoya). The total number of MSI/group were WT uninfected n = 296, WT infected n = 372, KO uninfected n = 373, KO infected n = 337. **(D-I)** Data were quantified and plotted using InForm Tissue Analysis Software (Akoya) for **(D)** Total cell number; **(E)** MHC II + cells; **(F)** CD206+ cells; **(G)** CD206+/MHCII+ cells; **(H)** MHCII+/PA+ cells; and finally **(I)** cells containing PA. WTU- wild-type/uninfected; WTI- wild-type/infected; TRAPKOU- *Trap5^-/-^/*uninfected; TRAPKOU- *Trap5^-/-^/*infected. Major histocompatibility complex II (MHC II); Cluster of Differentiation 206 (CD206); PA (*P. aeruginosa*). Statistical analysis was conducted using one-way ANOVA with a Dunnet’s *post hoc* test (**P<0.01; ***P<0.001; ****P<0.0001).

Determination of immune cell recruitment within the murine lung was determined by whole lung scans of wild-type and Trap5^-/-^ mouse lungs following H&E staining (**Figure 4A** and **Supplementary Figure 6**). Higher magnification imaging revealed increased immune cell recruitment in infected mouse lungs of both WT and Trap5^-/-^ mice. To further assess differences, TSA multiplex staining was utilized to determine the role of immune cell recruitment in bacterial clearance. Phenotype mapping shows the antibodies utilized in this study, with DAPI, MHCII (dendritic cells and macrophages), CD206 (inflammatory macrophages), and PA measured in each lung section (**Figure 4B**). Multispectral images (MSI) were obtained from 9 animals in each group (**Figure 4C**) using Vectra 3 Automated Quantitative Pathology Imaging System (Akoya). The total number of MSI/group were WT uninfected n = 296, WT infected n = 372, KO uninfected n = 373, KO infected n = 337. Importantly, MHC II^+^/PA^+^ cells were significantly increased in Trap5^-/-^ murine lung sections despite an increased number of CD206+ and MHCII+ cells (**Figures 4D–I**).

### TRAP5 significantly reduces innate NF-κB driven cell recruitment

**Figure 5:**
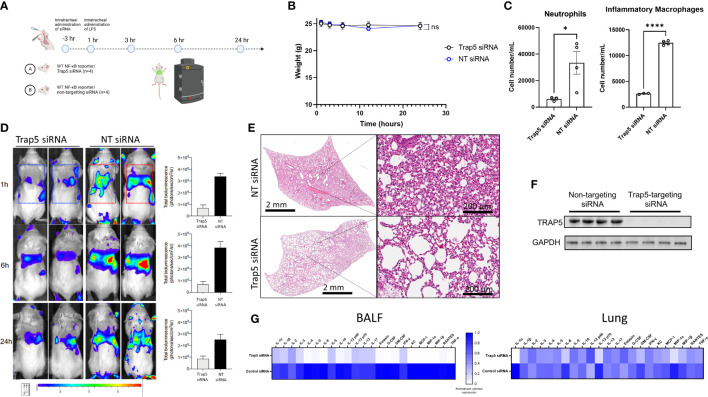
Intratracheal administration of Trap5 siRNA decreases the NF-κB response. **(A)** Experimental layout showing time points at which siRNA and LPS were administered to mice (n=4 per group). **(B)** Murine weights remained consistent throughout the course of the experiment with some transient weight loss recorded in the NT siRNA group. **(C)** Murine BALF neutrophils (live dead^+^/Ly6G^+^/CD11b^+^) and inflammatory macrophages (live dead^+^/Ly6G^-^/CD11b^+^/CD11c intermed) were measured using flow cytometry. Statistical analysis was performed using student’s *t*-test. *P  <  0.05 and ****P < 0.0001. **(D)** Noninvasive *in vivo* bioimaging of NF-κB reporter gene expression was performed using the IVIS Spectrum system. Representative images show bioluminescence at 1, 6, and 24 h after intratracheal LPS administration. Data is presented as a bar chart depicting measured bioluminescence intensity emitted from lung region of interest (ROI). Dotted squares represent the region of interest for data analysis. Data are presented as mean ± SEM (*n* = 4 mice per group). **(E)** Representative hematoxylin & eosin staining of murine lungs. Statistical analysis was performed using student’s *t*-test (*P  <  0.05; ****P < 0.0001). **(F)** Immunoblotting of murine lung homogenate. **(G)** Heatmap of murine BALF and lung cytokines from 23-plex analysis (cytokine values were mean normalized; high values-blue; low values-white).

To further investigate the mechanism by which TRAP5 inhibits cell recruitment, BALB/c-Tg (Rela-luc)31Xen mice were intratracheally (i.t.)-administered TRAP5-targeting siRNA and monitored for 24h following i.t. administration of LPS (**Figure 5A**). Murine weight was largely maintained throughout the experiment, with the non-targeting (NT) siRNA group displaying increased weight loss at the 10h time point (**Figure 5B**). Cell recruitment was measured in BALF, with significant increases in neutrophils and inflammatory macrophages seen in the NT group compared to the TRAP5 siRNA group (**Figure 5C**). These results were supported by IVIS monitoring of *in vivo* NF-κB production, with TRAP5 siRNA-administered mice displaying significantly reduced NF-κB signal within the lung at all time points post-LPS administration (**Figure 5D**). Histological analysis of murine lung tissue indicated decreased cell recruitment and decreases in lung damage in the TRAP5 siRNA group compared to the NT siRNA group (**Figure 5E** and **Supplementary Figure 7**). Immunoblotting of murine lung homogenates confirmed total reductions in TRAP5 protein production following siRNA knockdown (**Figure 5F**). This was further examined using multiplex cytokine measurement in murine lung homogenates, with significant downregulation of chemotactic cytokines in the BALF of the TRAP5 siRNA group compared to the NT siRNA control (**Figure 5G**). Conversely, fewer reductions in lung cytokines were seen in the TRAP5 siRNA group compared to the NT siRNA group indicative of a higher level of cytokines compared to the BALF in Trap5^-/-^ animals (**Figure 5G** and **Supplementary Figures 8**-**10**).

### Functional TRAP enzyme activity drives immune cell recruitment, promoting bacterial killing

**Figure 6:**
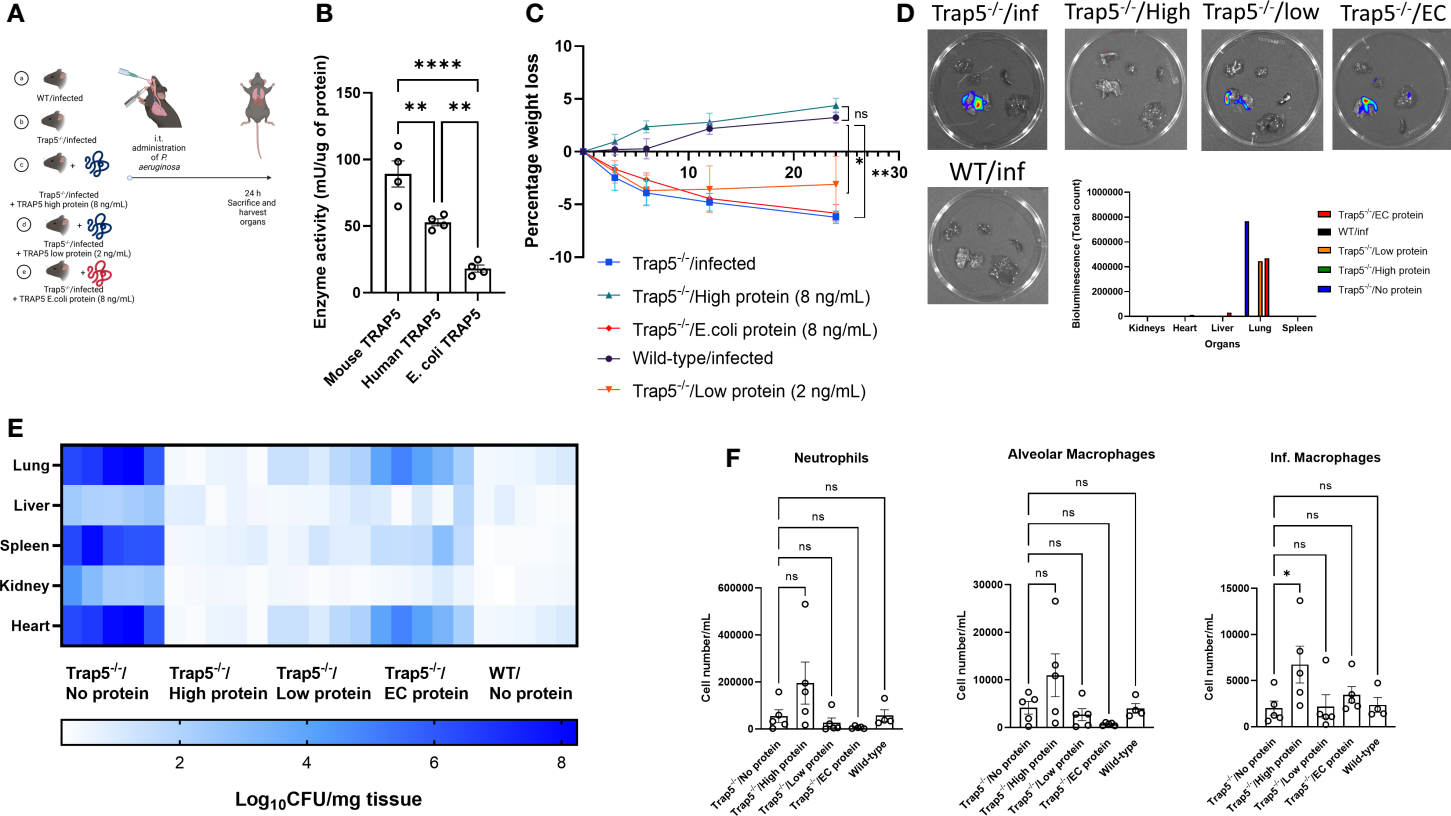
Functional TRAP5 protein rescues TRAP5 knockout animals from *Pseudomonas aeruginosa* lung infection. **(A)** Experimental layout showing time points at which bacteria were administered to mice, with proteins administered 30 min before infection (n=5 per group). **(B)** Commercially available TRAP5 proteins were tested for their enzymatic activity using a phosphatase activity assay. Statistical analysis was conducted using one-way ANOVA with a Dunnet’s *post hoc* test (**P <0.01; ****P < 0.0001). **(C)** Following i.t. administration of different proteins and bacteria, mice were monitored for weight loss over 24 h. Statistical analysis was performed using student’s *t*-test (*P  <  0.05; **P < 0.01). **(D)** Mice were euthanized and organs were imaged using the IVIS Spectrum system, with total bioluminescence counts in the figure inset. **(E)** Confirmatory bacterial plating was conducted on murine lung, liver, spleen, kidney, and heart tissues. **(F)** Flow cytometry of murine BALF revealed differential immune cell recruitment following TRAP5 protein administration. Statistical analysis was conducted using one-way ANOVA with a Dunnet’s *post hoc* test (*P <0.05). NS-not significant.

Finally, to determine whether enzymatic activity of TRAP5 played a direct role in immune cell recruitment and bacterial killing we investigated whether intratracheally-administered TRAP5 protein could resolve airway infection in Trap5^-/-^ mice. Based on *in vivo* mouse TRAP5 lung concentrations reported above (**Figure 2I**), we administered recombinant murine TRAP5 (expressed in eukaryotic cells) as well as human recombinant TRAP5 (expressed in *E. coli*) proteins to *P. aeruginosa-*infected Trap5^-/-^ mice (**Figure 6A**). Commercially produced TRAP5 proteins were tested for their enzymatic activity (**Figure 6B**), with *E. coli* produced enzyme displaying significantly lower activity. Murine weight loss was significantly attenuated following administration of 8 ng/mL of TRAP5 protein compared to the Trap5^-/-^ control group, with very little change in murine weight in the low protein and *E. coli* protein groups (**Figure 6C**). Bacterial bioluminescence was again increased in the Trap5^-/-^ infected, Trap5^-/-^ infected/low protein, and Trap5^-/-^ infected/*E. coli* groups with non-detectable levels in the Trap5^-/-^ infected/high protein and WT infected groups (**Figure 6D**). Confirmatory bacterial plating showed the Trap5^-/-^ infected group again displayed the highest bacterial burden in all tested organs (**Figure 6E**). Conversely, the Trap5^-/-^ infected/high protein group displayed similar bacterial organ counts as the WT infected group, providing compelling evidence for the crucial role of TRAP5 in bacterial clearance. Furthermore, the decreased enzymatic activity offered by TRAP5 protein expressed in *E.coli* decreased levels of protection from bacterial infection, with slightly more protection provided by low TRAP5 protein concentration. Finally, murine BALF was assessed for immune cell recruitment with neutrophils, inflammatory macrophages, and alveolar macrophages increased following the addition of high TRAP5 protein concentrations (**Figure 6F**). Conversely, no increases in immune cell recruitment were seen for the Trap5^-/-^ low protein, Trap5^-/-^ *E. coli* protein, or Trap5^-/-^ infected groups.

## Discussion

In this study, we demonstrate an integral role for tartrate resistant acid phosphatase 5 (TRAP5) in the airway inflammatory response. We show evidence for TRAP5 involvement in activation of NF-κB *via* expression of proinflammatory cytokines/chemokines and the recruitment of immune cells that subsequently amplify inflammation, upon bacterial infection and challenge with LPS.

**Figure 7:**
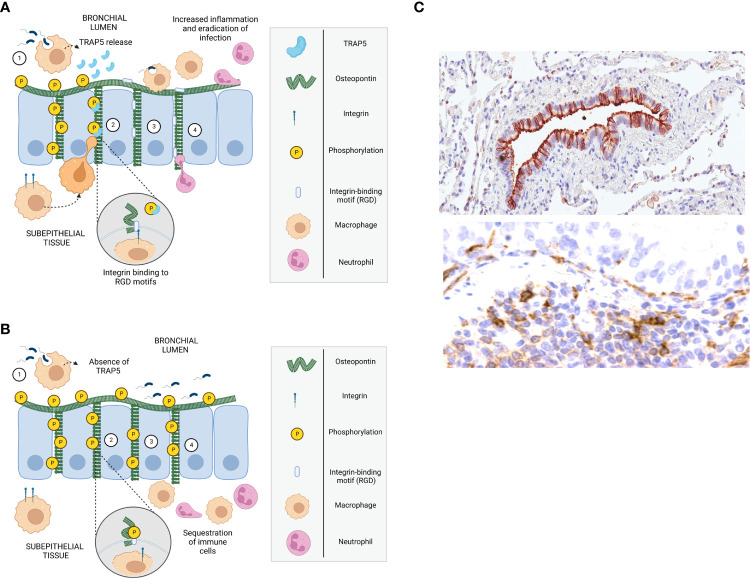
Mechanism describing TRAP5/OPN-dependent recruitment of immune cells to the airways. **(A, B)** Schematic drawings of mechanism describing immune cell recruitment, with **(A)** describing the development of inflammation in infected wild-type mice and **(B)** in Trap5-/- mice. 1. Residential alveolar macrophages detect bacteria through pathogen recognition receptors (TLRs) and become activated, stimulating epithelial cells that in turn secrete cytokines/chemokines, attracting neutrophils and macrophages. 2. TRAP5 released (absent in Trap5-/- mice) by activated alveolar macrophages dephosphorylates osteopontin (OPN) exposing integrin-binding motifs (i.e. regions containing RGD and RGD-like sequences). 3. Immune cells (e.g. inflammatory macrophages and neutrophils) migrate into the bronchial lumen in a haptotactic manner. The migration of immune cells is dependent on their integrins binding to the RGD-motifs of OPN, which is affected by the phosphorylation status of OPN (as shown inset). In the absence of TRAP5 (as seen in Trap5-/- mice), OPN remains phosphorylated and immune cells accumulate in the subepithelial tissue. 4. Recruited immune cells further amplify inflammation by release of cytokines/chemokines attracting more immune cells to the lumen to clear bacterial infections. **(C)** OPN staining in human lung tissue shown in the top figure (brown), where it is localized on the apical surface as well as in the intercellular space between the bronchial epithelial cells of a small airway. TRAP5 and released TRAP5 detected in macrophages of subepithelial tissue of the airways (bottom panel).

Previous work has shown that, in a model of intraperitoneally administered *Staphylococcus aureus*, TRAP-deficient animals had significantly delayed bacterial clearance due to assumed macrophage recruitment deficiencies (12). However, no further analyses were conducted to elucidate these findings. An important discovery in the current study is the impaired recruitment of neutrophils as well as alveolar and inflammatory macrophages to the BALF, providing a key contribution to the vulnerability towards bacterial infection. Possible explanations for this lack of recruitment could be the role of TRAP5 as a phosphatase against phosphotyrosine‐containing peptides and the phosphoserines of OPN (36) as graphically depicted in **Figure 7**.

OPN-deficient mice have impaired recruitment of neutrophils to the airways during Gram-negative airway infection, with add-back of OPN to OPN^-/-^ mice increasing neutrophil recruitment (37, 38). Similarly, in this work, add-back of functional TRAP5 to the murine lung served to increase immune cell recruitment and facilitated bacterial clearance. Moreover, this is supported by work in osteoclast migration, whereby TRAP5b was highlighted as the main contributing isoform to migration, as elucidated by small-molecule perturbation (19, 39).

Furthermore, a cytokine gradient was seen between the BALF and lung tissue in Trap5^-/-^ mice, causing potential differences in immune cell recruitment between these two sites, favoring an immune cell-rich lung environment. KC and IL-6 have been shown in murine precision cut lung slices (PCLS (40);) infected with *P. aeruginosa* to regulate early recruitment of neutrophils (41, 42) and continued pro-inflammatory actions (43, 44), respectively. This data further supports the role of TRAP5 in immune cell recruitment and should be supplemented with future studies using precision-cut lung slices (PCLS) from siRNA knockdown or Trap5 knockout animals. Upregulation and activation of membrane-trafficking regulators and lysosomal enzymes in innate phagocytes, including TRAP5, are crucial for the efficient killing of internalized bacteria (45–47) and could further explain the lack of killing observed during lung infection.

OPN/TRAP5 interactions may act as a both a direct chemoattractant and a matrix where different integrins bind an RGD-motif and an encrypted RGD-like motif during cell migration (27). However, the interaction of migrating immune cells with OPN is complex. Serine phosphorylation of OPN on specific sites is required for functional interaction with integrins but not CD44 receptors (28). Dephosphorylation of OPN by molecules such as TRAP5 exposes integrin-binding motifs, increasing the affinity of αvβ3 (48, 49), regulating cell spreading, adhesion, and migration. In the current study, we were not able to characterize posttranslational modifications of OPN-fragments using mass spectrometry. However, we could confirm and highlight the large number of phosphorylation sites on recombinant OPN, expressed in eukaryotic cells, utilizing this technique.

Taken together, the findings of this study suggest a key role for TRAP5 in NF-κB-driven airway inflammation and host defense. Precise molecular mechanisms surrounding TRAP5 as a key hub during inflammation warrants further investigation. These findings could also be used as a template for future development of TRAP5-targeting therapies in states of disease characterized by long-standing and excessive inflammation as seen in CF and COPD.

## Data availability statement

The data presented in the study are deposited in the ProteomeXchange repository, accession number PXD038135.

## Ethics statement

Lung tissue was obtained after written informed consent, approval by the Regional Ethical Review Board in Lund (approval no. LU412-03) and at Karolinska Institutet, Solna (95-347), and performed in accordance with the Declaration of Helsinki as well as relevant guidelines and regulations. The patients/participants provided their written informed consent to participate in this study. All animal experiments were approved by the Malmö/Lund Animal Care Ethics Committee (M3802-19).

## Author contributions

The study was conceptualized and designed by LT and AE. Experiments were performed by LT, JB, RB, MP, PL, MNA, CW, and LP. Data analysis was performed by LT, RB, MP, PL, JB, MNA, CW, PÖ, JE, LP, GA, and AE. LT drafted the initial manuscript with all authors contributing to editing and discussion of results. AE, GA, and LT contributed funding to the study. All authors contributed to the article and approved the submitted version.
