# Supplementary material for: Tartrate resistant acid phosphatase 5 (TRAP5) mediates immune cell recruitment in a murine model of pulmonary bacterial infection
Source: Front Immunol. 2022 Dec 8;13:1079775. doi: 10.3389/fimmu.2022.1079775 (PMC9779928; doi:10.3389/fimmu.2022.1079775)
Supplement: Supplementary file 1 [file DataSheet_1.docx]

Supplementary Material


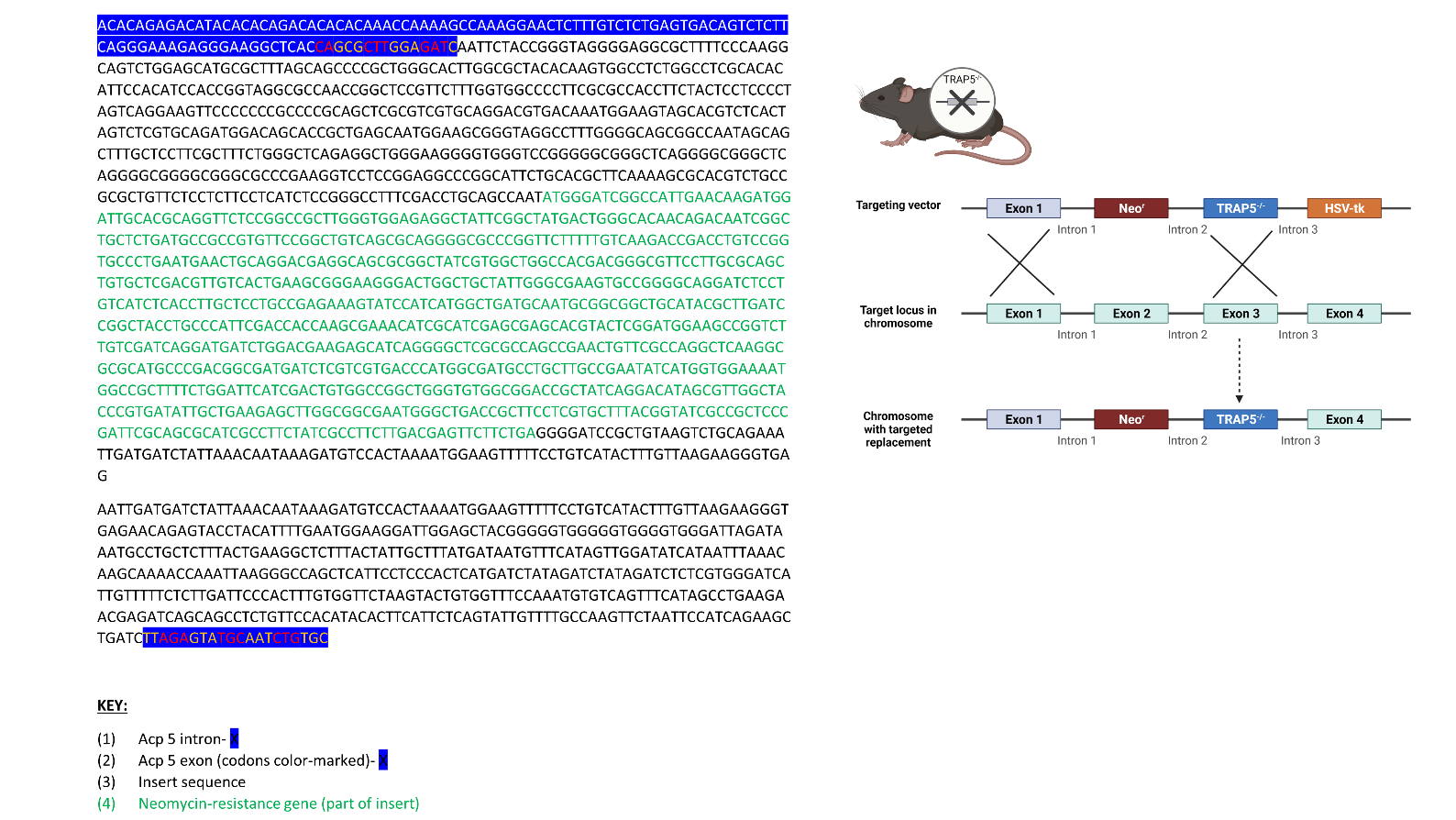


**Supplementary Figure 1: Whole genome sequencing (WGS) of Trap5^-/-^ mice.** Insertion of the resistance sequence is shown alongside.


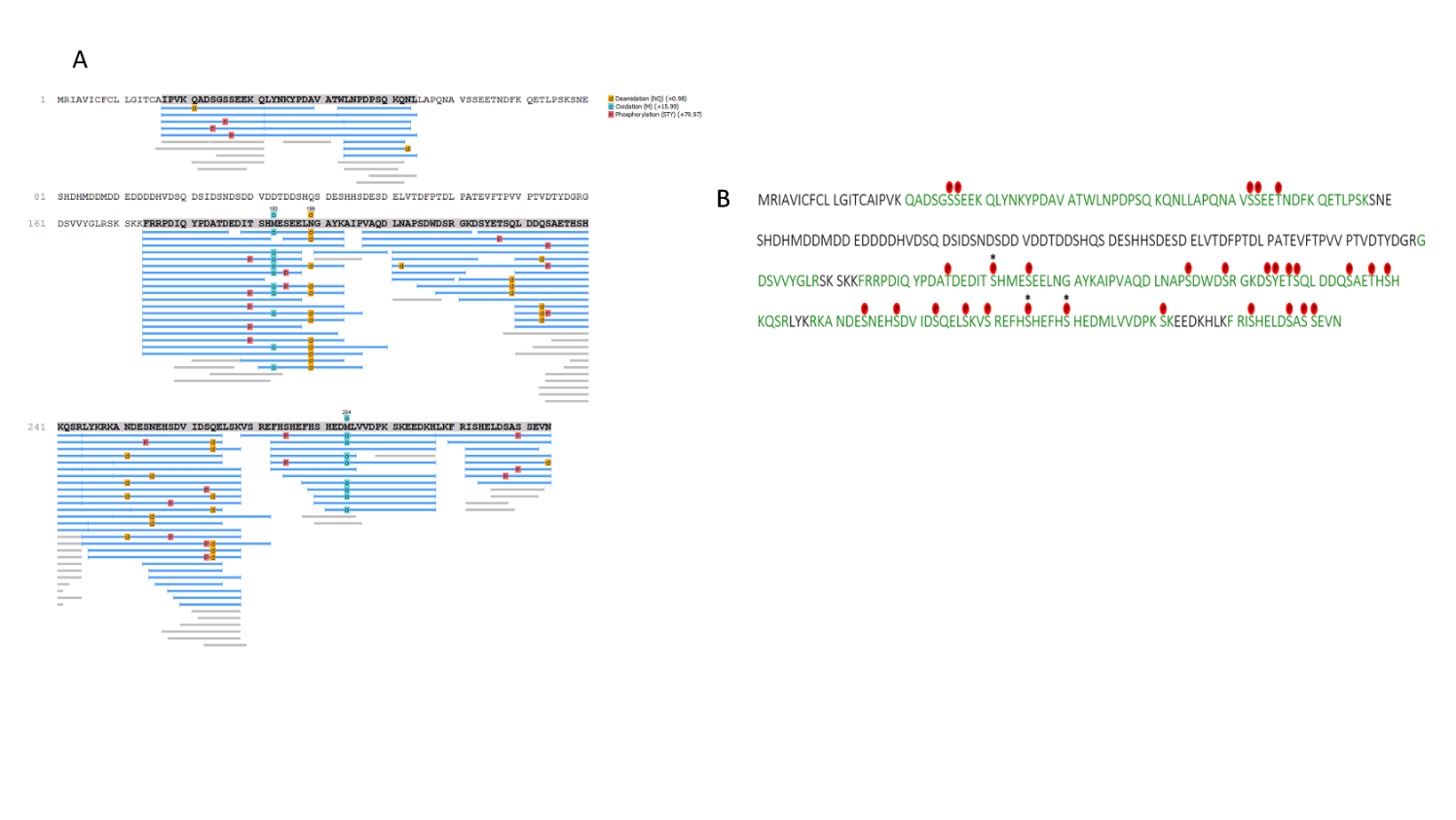


**Supplementary Figure 2: Phosphopeptides and non-phosphorylated peptides detected in HEK293-expressed osteopontin using mass spectrometry.** (A) Blue lines: high confidence MS/MS spectra; Gray lines: *de novo* MS/MS spectra. The total sequence coverage was 63% with 19 phosphopeptides found. (B) Localization of phosphorylated sites on threonines and serines in HEK293 derived OPN. The total sequence coverage is noted as green peptides. Symbols: known phosphorylation site (red ‘P’), new phosphorylation site discovered (red ‘P*’).


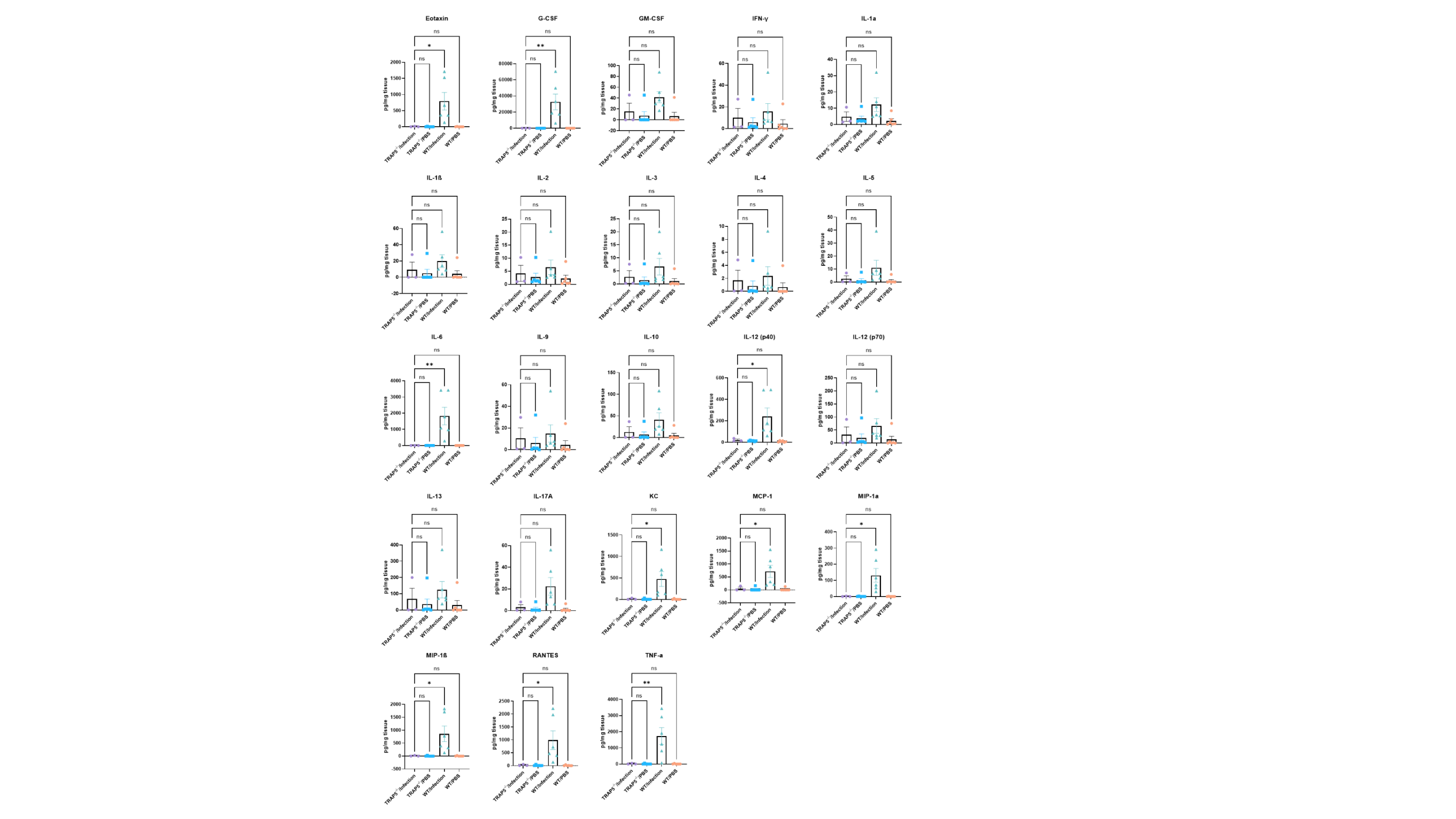


**Supplementary Figure 3: Multiplex immunoassay (Bio-plex) data for BALF from Trap5^-/-^ and wild-type (WT) mice following *P. aeruginosa* infection.** Analysis was conducted using one-way ANOVA with a Dunnet’s post hoc test (**P<*0.05*; **P<*0.01*; ***P<*0.005*; *****P<*0.001).

**
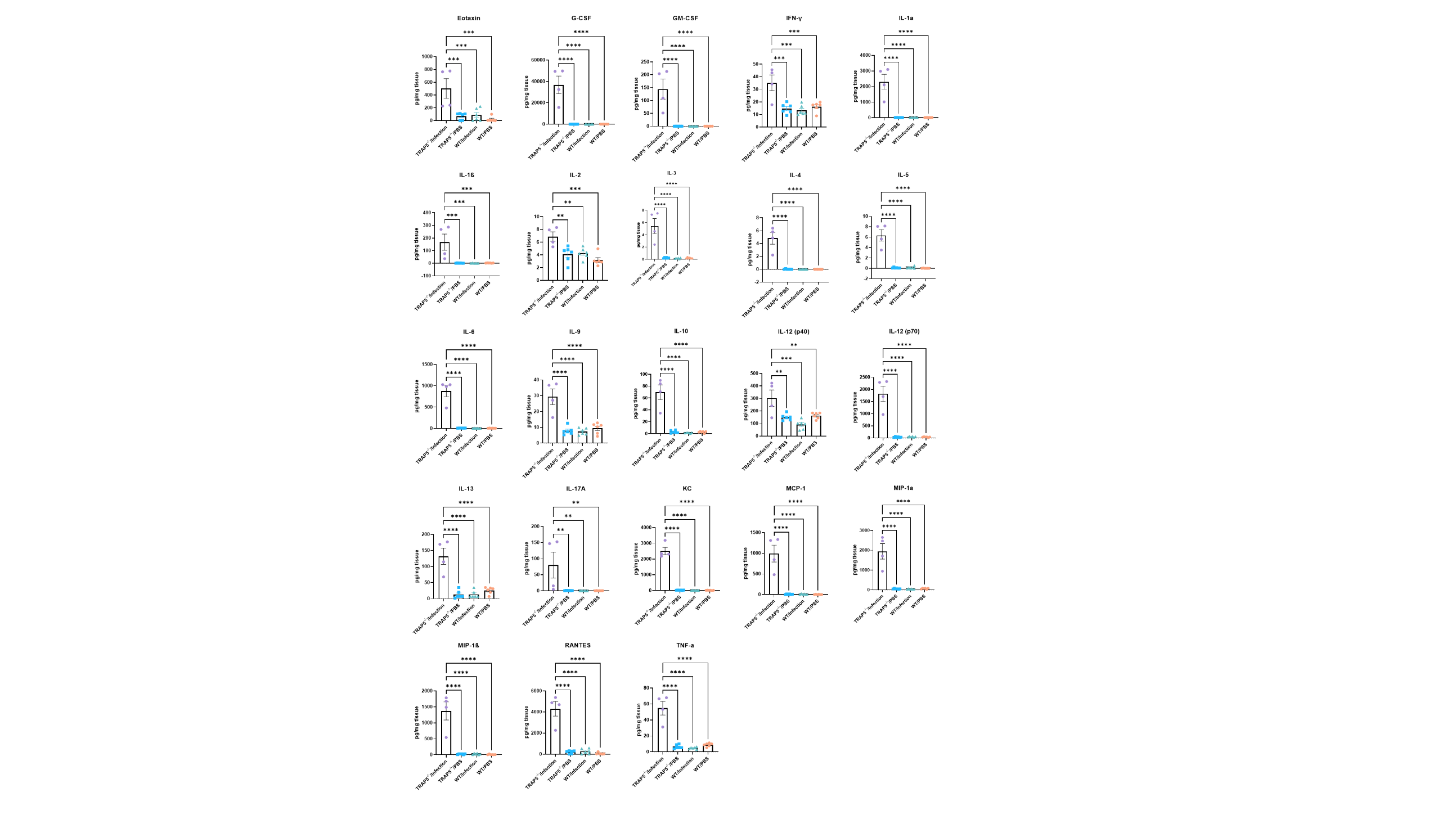
**

**Supplementary Figure 4: Multiplex immunoassay (Bio-plex) data for lung tissue from Trap5^-/-^ and wild-type (WT) mice following *P. aeruginosa* infection.** Analysis was conducted using one-way ANOVA with a Dunnet’s post hoc test (**P<*0.05*; **P<*0.01*; ***P<*0.005*; *****P<*0.001).


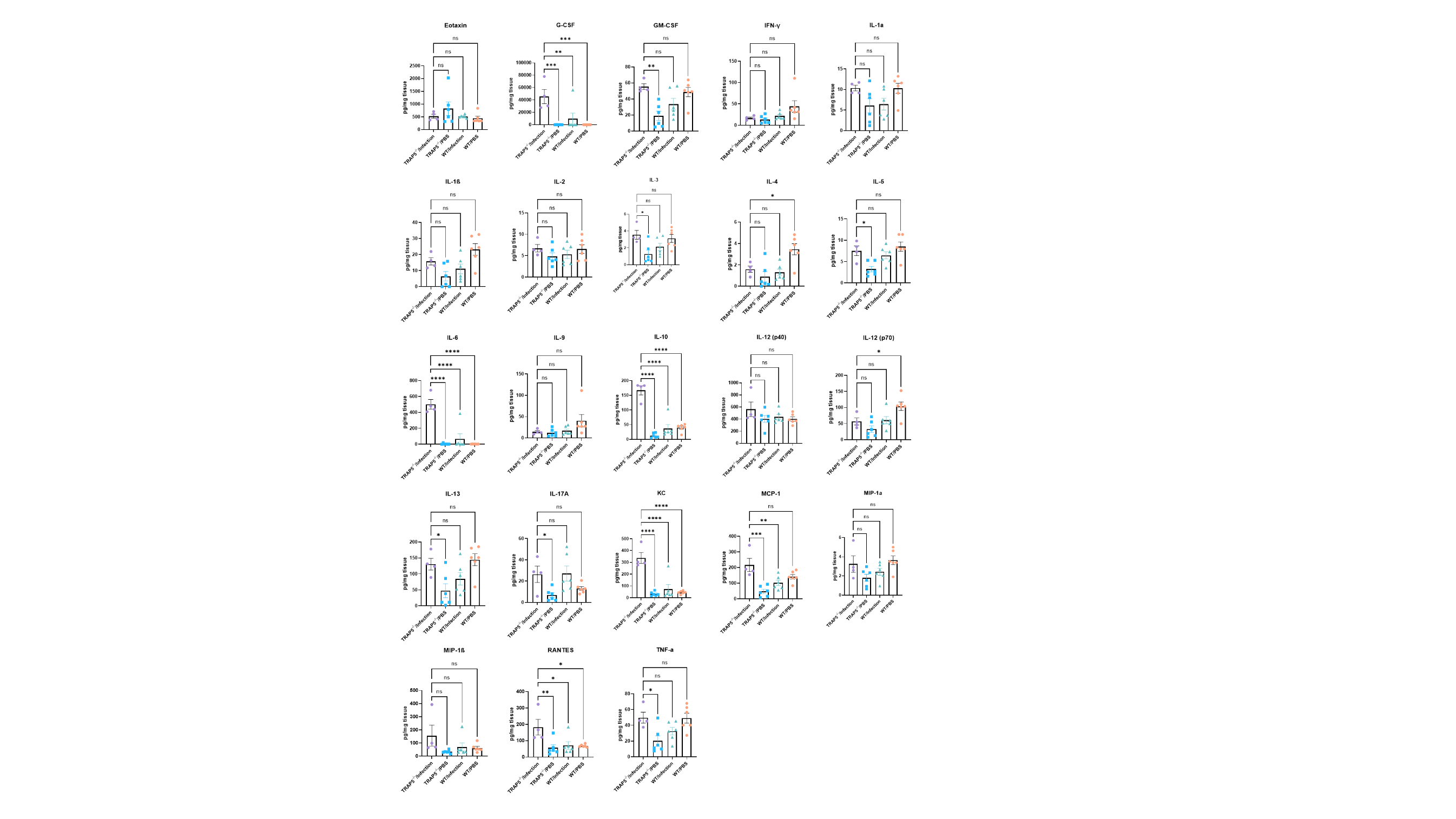


**Supplementary Figure 5: Multiplex immunoassay (Bio-plex) data for plasma from Trap5^-/-^ and wild-type (WT) mice following *P. aeruginosa* infection.** Analysis was conducted using one-way ANOVA with a Dunnet’s post hoc test (**P<*0.05*; **P<*0.01*; ***P<*0.005*; *****P<*0.001).

**
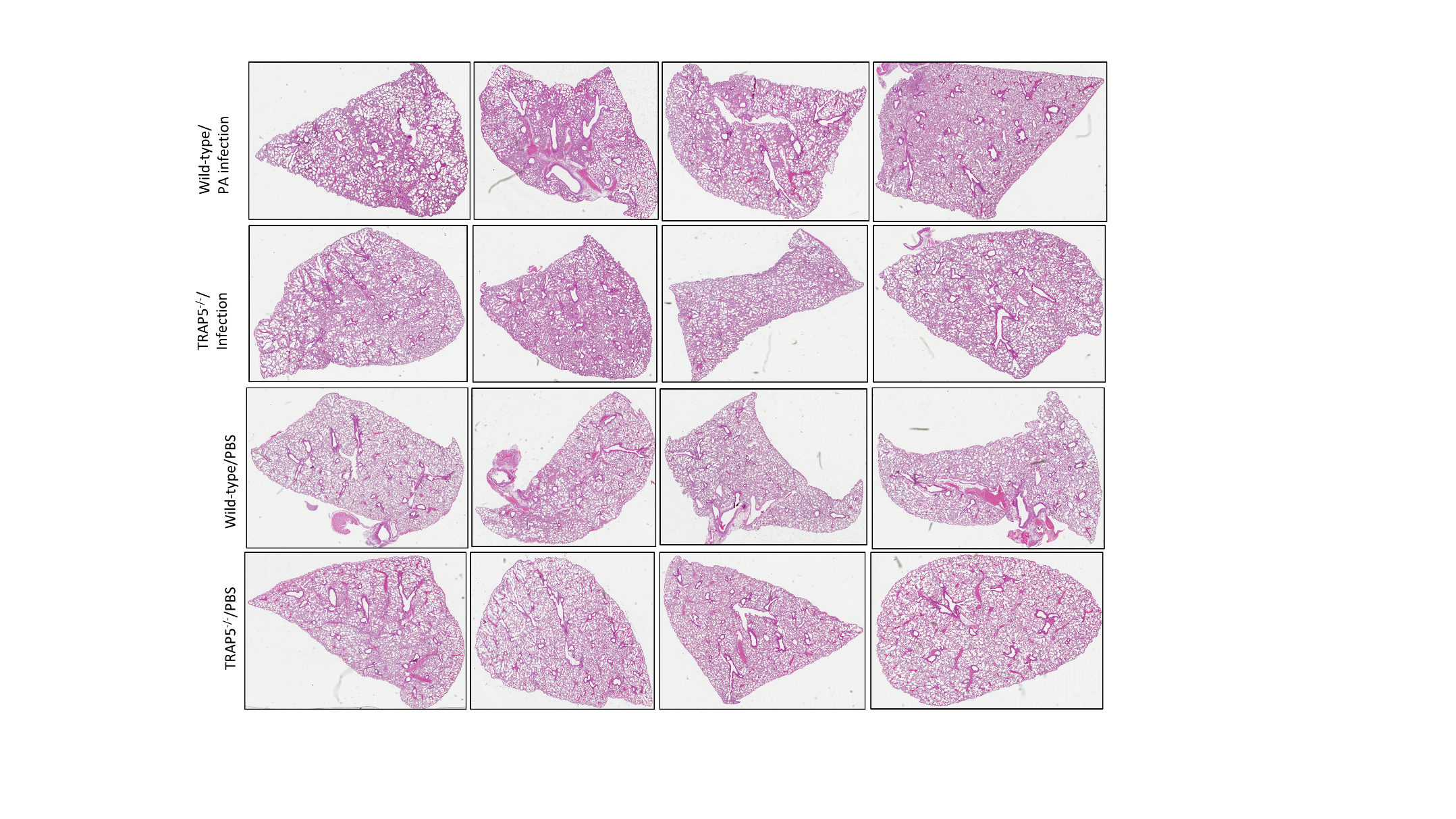
 Supplementary Figure 6: Whole lung scans from murine lungs from *P. aeruginosa* experiments stained with H&E.** Scale bar = 2 mm.


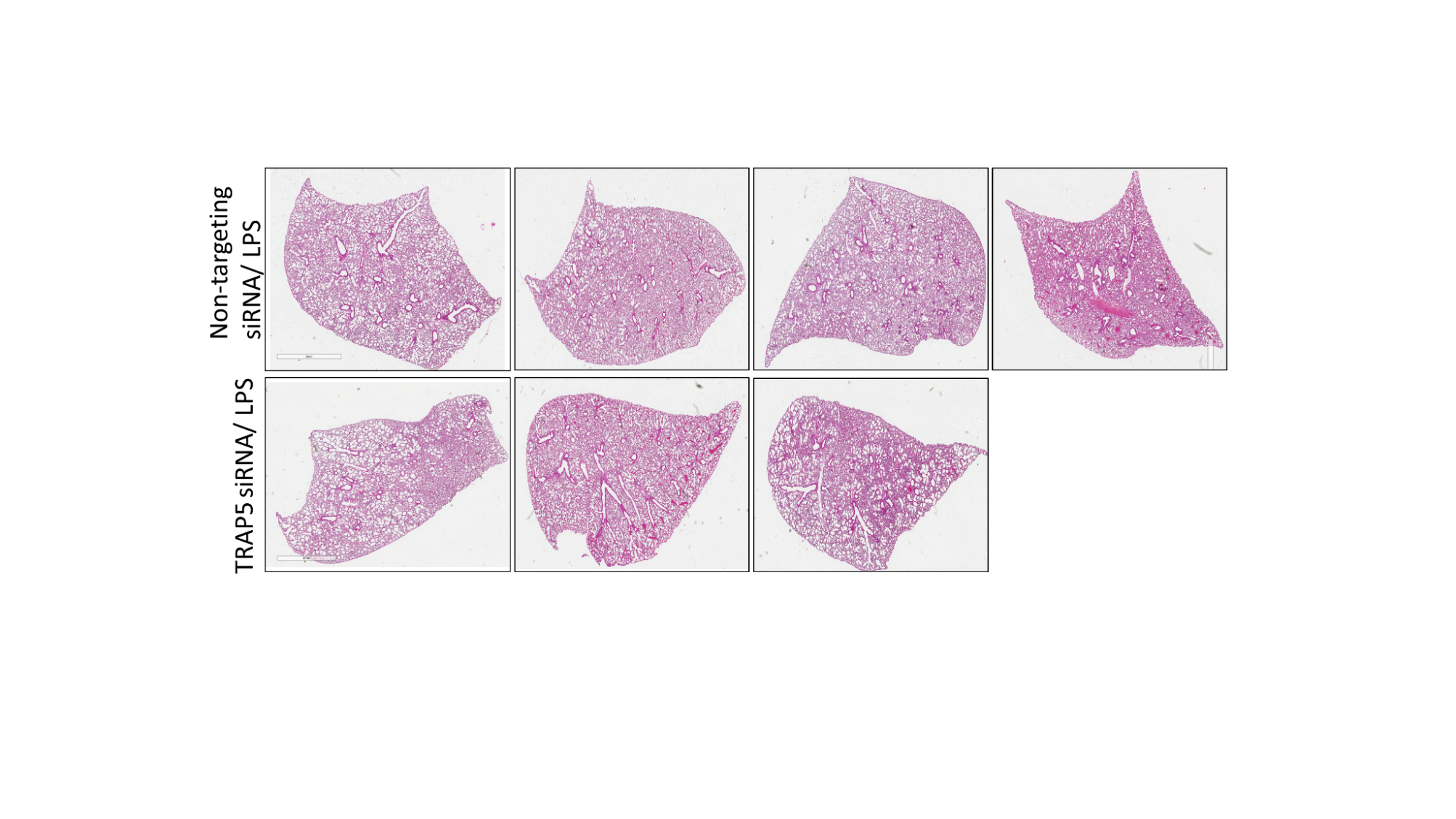


**Supplementary Figure 7: Whole lung scans from murine lungs from BALB/c-Tg(NF-κB-RE-Luc)-Xen reporter experiments stained with H&E.** Scale bar = 2 mm.


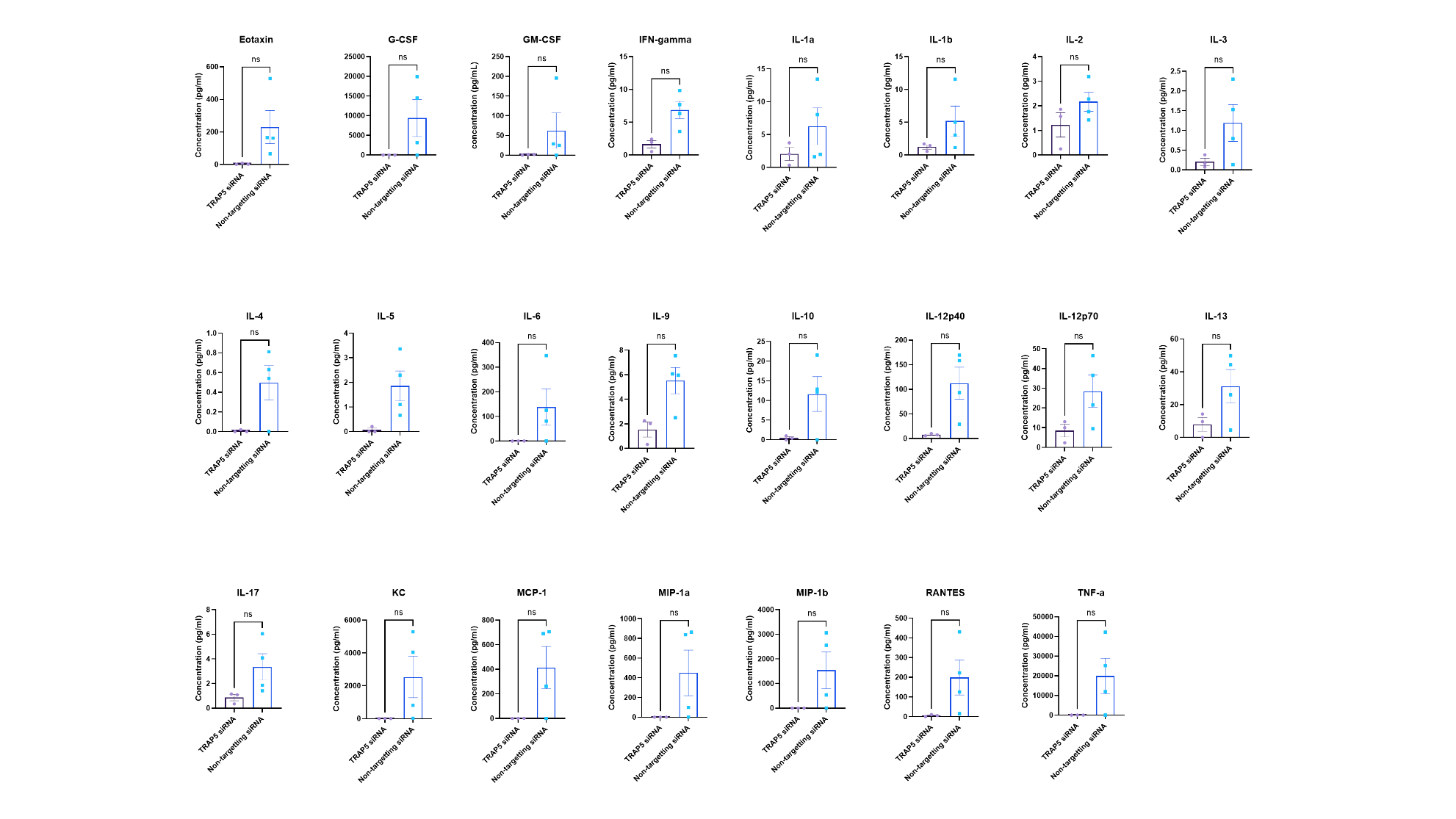


**Supplementary Figure 8: Multiplex immunoassay (Bio-plex) data for BALF from BALB/c-Tg(NF-κB-RE-Luc)-Xen reporter mice (knocked down with Trap5- or non-targeting siRNA) and stimulated with LPS.** In experiments using two groups, results were compared using unpaired *t* test with Welch’s correction.

**
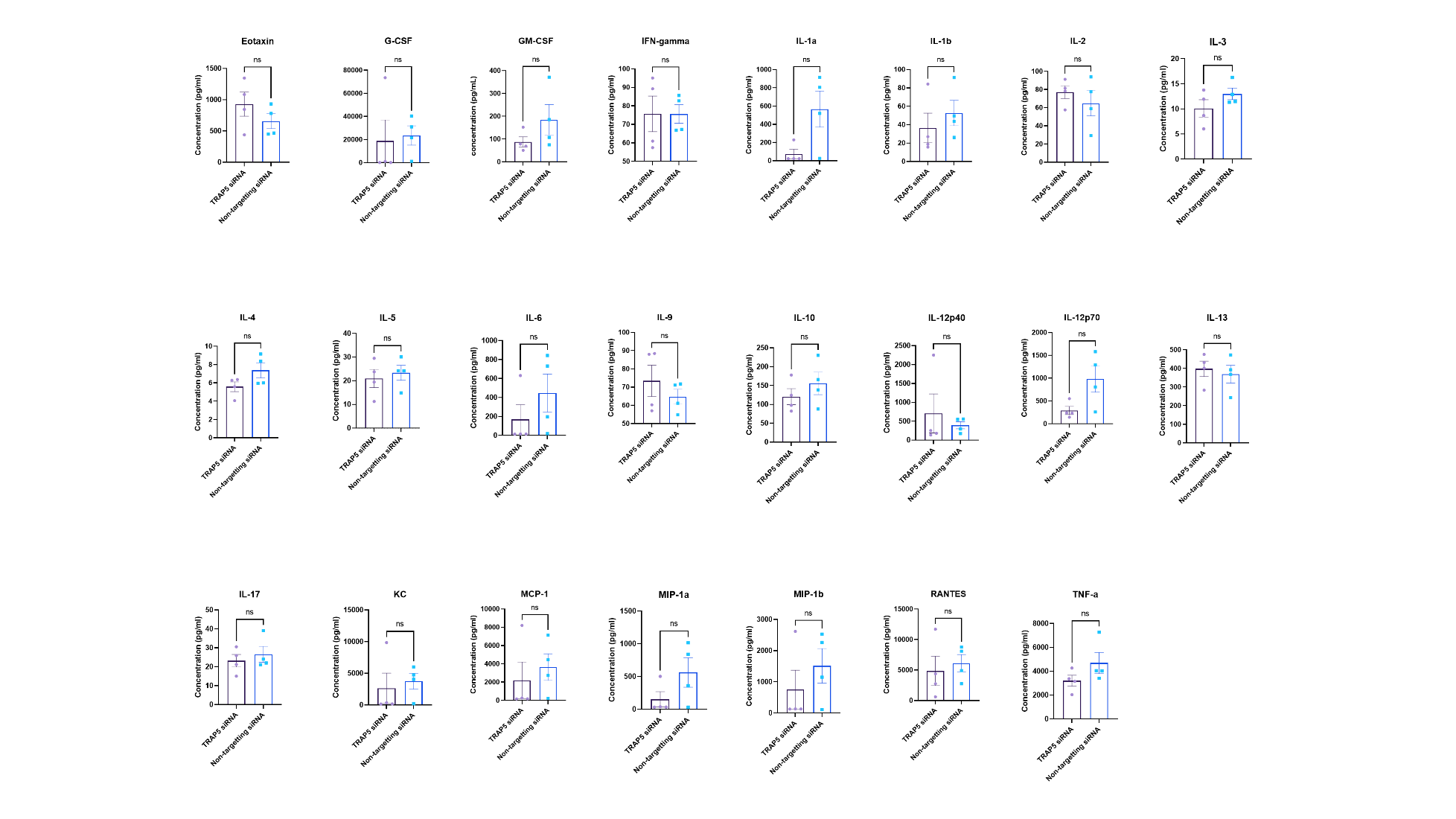
**

**Supplementary Figure 9: Multiplex immunoassay (Bio-plex) data for lung tissue from BALB/c-Tg(NF-κB-RE-Luc)-Xen reporter mice (knocked down with Trap5- or non-targeting siRNA) and stimulated with LPS.** In experiments using two groups, results were compared using unpaired *t* test with Welch’s correction.

**
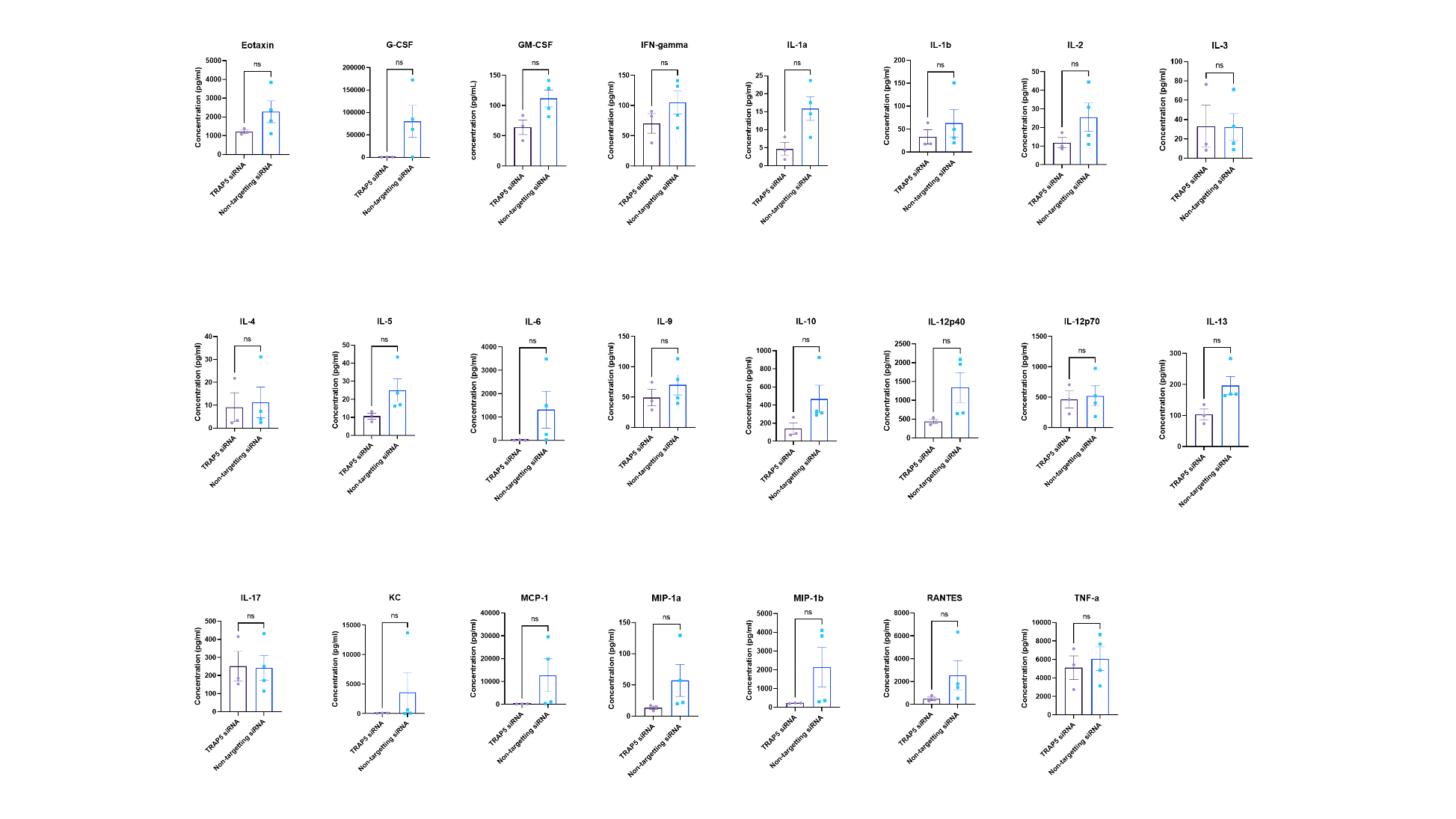
**

**Supplementary Figure 10: Multiplex immunoassay (Bio-plex) data for plasma from BALB/c-Tg(NF-κB-RE-Luc)-Xen reporter mice (knocked down with Trap5- or non-targeting siRNA) and stimulated with LPS.** In experiments using two groups, results were compared using unpaired *t* test with Welch’s correction.

| **Supplemental Table 1. Characteristics of patients with CF and controls** | | |
| --- | --- | --- |
|  | **Controls** | **CF** |
| Sex (M/F, n) | 2/6, 8 | 2/3, 5 |
| Age^a^ (years) | 63 (33–76) | 30 (23–38) |
| Current smokers (y/n) | 0 | 0 |
| Ex-smokers (y/n) | 0 | 0 |
| Inhaled GCS (y/n) | 0 | 5/0 |
| Oral GCS (y/n) | 0 | 3/2 |
| β_2_-agonist (y/n) | 0 | 5/0 |
| *Lung function* |  |  |
| FEV_1_ % of predicted^a^ | 110 (82–141) | 31 (22–45) |
| FEV_1_/(F)VC^a^ | 86 (66–121) | 50 (33–84) |
| VC % of predicted^a^ | 104 (82–126) | 54 (46–70) |
| TLC % of predicted^a^ | ND | 104 (79–129) |
| RV % of predicted^a^ | ND | 215 (113–318) |
| % TLCO SB | ND | 60 (50–71) |
| ^a^ Data are given as mean (range). M = male, F = female, GCS = glucocorticosteroid, FEV1 = forced expiratory volume in 1 second, VC = vital capacity, TLC = total lung capacity, RV = residual volume, TLCO = diffusing capacity for carbon monoxide. | | |
